# Effectiveness of a web-based intervention in reducing binge drinking among nightclub patrons

**DOI:** 10.11606/S1518-8787.2018052000281

**Published:** 2018-01-16

**Authors:** Yago C Baldin, Adriana Sanudo, Zila M Sanchez

**Affiliations:** I Universidade Federal de São Paulo. Escola Paulista de Medicina. Departamento de Medicina Preventiva. São Paulo, SP, Brasil

**Keywords:** Binge Drinking, prevention & control, Internet, Evaluation of the Efficacy-Effectiveness of Interventions, Randomized Controlled Trial, Bebedeira, prevenção & controle, Internet, Avaliação de Eficácia-Efetividade de Intervenções, Ensaio Controlado Aleatório

## Abstract

**OBJECTIVE:**

To evaluate the effectiveness of a web-based intervention in reducing binge drinking among nightclub patrons after six months.

**METHODS:**

We carried out a website survey with probabilistic sample in 31 nightclubs in the city of São Paulo, Brazil, which originated a randomized controlled trial with 1,057 participants. Those classified as problem drinkers (n = 465) using the Alcohol Use Disorders Identification Test were randomized into two study groups – intervention and control. The web-based intervention consisted of exposing the participants to a normative feedback screen about their alcohol consumption, characterizing the risks associated with amount consumed, money spent on drinks, drinking and driving, risk classification of Alcohol Use Disorders Identification Test, and tips to reduce damage.

**RESULTS:**

There was a significant reduction in the practice of binge drinking in the week estimated at 38% among participants in the intervention group after six months (p < 0.05). However, there was no significant reduction in the outcomes when we analyzed the intervention and control groups and at baseline and after sixth months, simultaneously.

**CONCLUSIONS:**

We cannot conclude that digital tools reduce the pattern of binge drinking among party goers in São Paulo. More studies are needed with this methodology because of its attractiveness to this type of group, given the privacy and speed that personalized information is transmitted.

## RESUMO

**OBJETIVO:**

Avaliar, após seis meses, a efetividade de uma intervenção digital na diminuição da prática de *binge drinking* entre frequentadores de baladas.

**MÉTODOS:**

Foi realizado um inquérito de portal através de uma amostra probabilística em 31 baladas da cidade de São Paulo, a qual originou um ensaio controlado randomizado com 1.057 participantes. Aqueles classificados como uso problemático do álcool (n = 465) através do *Alcohol Use Disorders Identification Test* foram randomizados em dois grupos de estudo – intervenção e controle. A intervenção digital consistiu em expor os participantes a uma tela de *feedback normativo* sobre seu consumo de álcool, caracterizando os riscos associados à quantidade consumida, valores gastos com bebidas, beber e dirigir, classificação do risco do *Alcohol Use Disorders Identification Test* e dicas de redução de danos.

**RESULTADOS:**

Houve uma redução significativa da prática de *binge drinking* na semana estimada em 38% entre os participantes do grupo intervenção após seis meses (p < 0,05). Porém, não houve redução significativa nos desfechos quando se analisam os grupos intervenção e controle e os tempos início e seis meses após, simultaneamente.

**CONCLUSÕES:**

Não se pode concluir que as ferramentas digitais reduzem o padrão de *binge drinking* nos frequentadores de baladas paulistanas. São necessários mais estudos com esta metodologia devido ao caráter atrativo para esse grupo, tendo em vista a privacidade e a rapidez que as informações personalizadas são transmitidas.

## INTRODUCTION

Nightclubs are an important place for leisure and entertainment for young persons^1^. They are an environment where changes in social patterns are tolerated and pleasure is stimulated^2^ and, together with the abusive alcohol consumption, they contribute with the increased risk exposure of nightclub patrons, such as physical or sexual violence^3^, aggressions, and conduct violations^4^. Binge drinking (BD)^5^ is common in nightclubs, which can be defined as the consumption of at least four doses of alcohol in a single occasion for women and five doses for men^6^. This practice can increase the chance of harmful consequences from alcohol abuse^7^, as it is associated with higher chances of sexual abuse, suicide attempts, unprotected sex, unwanted pregnancies, drunkenness, falls, accidents, and inflammatory diseases^8^.

In order to reduce the consumption of alcohol and other drugs, recent web-based interventions (i.e., offered over the Internet) have been tested in developed countries, especially among young persons or students^9^ ^,^ ^10^, using personalized normative feedback messages^11^ ^,^ ^12^. Personalized messages address educational information about alcohol use, personal messages about the drinking profile of participants, risk factors and harmful consequences, costs associated with consumption, comparisons with other profiles, and tips for harm reduction^13^ ^,^ ^14^, by confronting individual consumption with population consumption or by providing ways on how to change behavior^15^. The advantage of this type of tool is that it can be used on a large scale because it is easy to access and has a low cost^16^, besides respecting the privacy of participants^17^.

International studies have shown a significant reduction in the practice of BD in university students after six months of personalized normative feedback via the Internet^18^ ^,^ ^19^. Kypri et al. have observed a significant reduction of 11% in the total volume of alcohol consumed after six months, reducing personal, sexual, and legal problems related to alcohol, and a significant reduction of 19% in relation to academic problems, such as failure to perform tasks because of alcohol consumption^20^ ^,^ ^21^.

Although Brazil has broad Internet access^22^, web-based interventions aimed at reducing alcohol abuse in the population have been used only recently^23^, even though it is clear that this type of tool must be implemented in the country following adaptations that take into account cultural differences^24^. Therefore, the introduction of interventions among party goers to reduce problems related to BD is a necessary and promising field of activity^25^, which makes this an innovative study. Thus, the objective of this study was to evaluate the effectiveness of a web-based intervention in reducing the practice of BD and its lack of control among nightclub patrons after six months.

## METHODS

### Sample of the Website Survey

The data used in this study originated from a website survey^26^ carried out to diagnose drug use and other risk behaviors among party goers in the city of São Paulo, Brazil, in 2013^27^ ^–^ ^29^. In this study, nightclubs were defined as any establishment that presented control of entry and exit, sale of alcoholic beverages, and dance floor. Details of the cross-sectional study are found in Sanchez et al.^28^

### Data Collection, Instruments, and Variables

**Figure 1:**
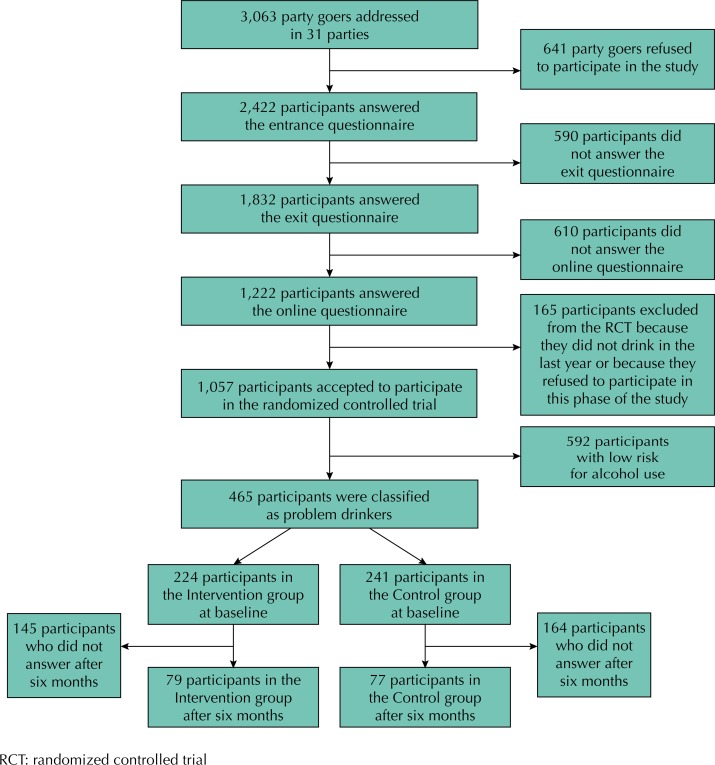
Flowchart for recruiting party goers and participation in the randomized controlled trial for the evaluation of a web-based intervention with normative feedback to reduce alcohol abuse. São Paulo, Brazil, 2013.

Figure 1 shows the flowchart for recruiting nightclub patrons, data collection, and participation in the intervention, from the beginning of the website survey to the sixth month of follow-up. The individuals selected in the queues of nightclubs participated in the study in three stages^26^: 1) in a face-to-face interview at the entrance and exit of the party, 2) answering an online questionnaire the next day, and 3) participating in the randomized controlled trial (RCT).

We systematically approached 3,063 subjects in the queues to enter 31 parties, so that the last one of every three individuals was invited to participate in the project. The inclusion criteria were: intention to enter the party and being at least 18 years old. In the case of refusal, data on age and sex were recorded and the next person was approached. A total of 2,422 subjects agreed to participate in the study (acceptance of 79%), who answered an interview about sociodemographic variables, patterns of alcohol consumption, and risk behaviors in parties in the 12 months before the interview; we also measured the alcohol concentration in the exhaled air at the time of the interview using a breathalyzer.

At the exit of the nightclub, those who had been interviewed at the entrance (identified via a bracelet with a unique numerical code) were asked to answer a new interview. Then, we gave them a folder with information about participation in an online research.

On the day after recruitment, we sent the link of a post-party questionnaire, subdivided into two modules, by e-mail to the 1,833 participants of the exit interview. Of these, 1,222 accessed the questionnaire (acceptance of 67%). The questionnaire included questions regarding the risk behaviors of participants after leaving the nightclub, alcohol expenses, higher amount of doses consumed, time of consumption, type of drink, and the evaluation of the Alcohol Use Disorders Identification Test (AUDIT), which has 10 questions producing a score ranging from zero to 40 that classifies the pattern of alcohol consumption into four risk levels (low risk use, risk use, harmful use, dependence).

The AUDIT has been developed by the World Health Organization (WHO) to identify the frequency, amount, and consequences of alcohol abuse^30^. As an evaluation tool, the AUDIT has shown an increasing number of evidence of being a rapid and successful methodology to identify these standards^31^. In this study, we used AUDIT to identify patterns of alcohol consumption among party goers and to select them for an RCT for a web-based intervention with personalized normative feedback.

In this study, we analyzed the following outcome variables: binge drinking in the month (BD_month_), binge drinking in the week (BD_week_), and lack of control over drinking behavior (lack of control); the last two come from the AUDIT and the first one is an extra question.

The BD_month_ refers to the following question (extra-AUDIT): “In the last four weeks, what was the highest amount of standard doses of alcohol you consumed on a single occasion?” This was an open question and we categorized the response of five or more doses as “yes” and the response of less than five as “no”.

The BD_week_ refers to question three of the AUDIT: “How often do you have five or more drinks on one occasion?”. We categorized as “yes” the response “Weekly” and “Daily or almost daily” and as “no” the response “Never”, “Less than monthly”, and “Monthly”. We highlight that we chose to use the option of five doses based on an average concentration of 12 g of ethanol per dose, following the original guidance of the AUDIT which presupposes 60 g of ethanol or more for this question. Thus, the dose options presented to the participant were 330 ml of beer (1 can; 4.7% ethanol / 130 ml wine; 12% ethanol / 40 ml distillate; 39% ethanol).

Lack of control over drinking behavior refers to question four of the AUDIT “How often during the last year have you found that you were not able to stop drinking once you had started?” We categorized as “yes” the responses “Less than monthly”, “Monthly”, “Weekly”, and “Daily or almost daily”, and as “no” the responses “Never”.

The sociodemographic data were taken from the initial database, that is, the one from the face-to-face interviews at the entrance of the nightclubs, as described in Santos et al.^32^ The sociodemographic adjustment variables used were: sex (male, female), age, and socioeconomic status, obtained from the Brazilian Association of Population Studies (ABEP, 2012)^33^ (A = high, B = medium-high, C = average, D = medium-low, E = low; the C, D, and E classes were grouped because of the small amount of sample).

### Randomized Controlled Trial Procedures

Of the 1,222 nightclub patrons who accessed the online questionnaire, a total of 1,057 had consumed alcohol in the last year and agreed to participate (acceptance of 86.5%) in the RCT. Allocation in the groups was randomized and stratified, performed on the website itself, using a stratified permuted block randomization algorithm, considering the following categories for each of the three randomization strata: 1) sex (female, male), 2) age group (18–24, 25–34, 35+), and 3) pattern of alcohol consumption (AUDIT classification in four categories, that is, 0–7, 8–14, 15–19, 20–40 points). At the end of the randomization, we obtained an intervention group with 515 (48.7%) participants and a control group with 542 (51.3%) participants.

After the initial evaluation, we applied the web-based intervention of personalized normative feedback, which followed the model adopted by Kypri et al.^19^ For the subjects of the intervention group, we exposed them to a customized normative feedback screen on the risks associated with alcohol consumption, consisting of four parts: 1) feedback on the level of alcohol consumed at the investigated moment with standardized information for each risk level (organic and mental health, as well as social complications), 2) information on social norms with bar graphs of the percentage of Brazilians, in the same age group and sex, who reported drinking less alcohol, emphasizing how this behavior is atypical for the general population (data from the general population of the household survey on alcohol of 2006^34^), 3) personalized estimate of financial expenses with alcohol per month and year, and 4) general information with data to minimize the adverse consequences of alcohol consumption. Participants of the control group received no intervention and only answered the questionnaire.

In this study, we included participants with scores greater than or equal to eight in the AUDIT, that is, those classified by the AUDIT as problem drinkers (AUDIT score ≥ 8). Of the 1,057 participants randomized in both groups, 465 (44.0%) met the inclusion criteria.

Six months after the initial response, all 465 participants were contacted by e-mail containing the link to a new questionnaire to be completed for a new evaluation. If they did not respond to the questionnaire within three days, we sent a new link, in addition to a SMS, informing them about the e-mail. After three e-mails sent without an answer, the participant was contacted by telephone and informed about the link sent. The logistics of sending e-mails, SMS, and later telephone calls was the same used in the different times of the study.

### Statistical Analysis

Qualitative variables were described as number (n) and percentage (%), while quantitative variables were described as mean and standard deviation. We evaluated the association between qualitative variables using Pearson's chi-square test or Fisher's exact test when one or more expected values were less than five. The comparison of the quantitative variables according to a dichotomous qualitative variable was performed using Student's t-test.

We analyzed the outcome variables using generalized linear models with panel data using the “xtlogit” procedure (Stata/SE 13.1 for Windows – StataCorp). All models included group effect (intervention or control), time (baseline or six months of follow-up), and group-time interaction. The interaction term allowed us to evaluate the effect of the intervention between the two evaluations. The results were presented as crude odds ratio (OR_c_) and respective 95% confidence interval (95%CI). In addition to this model, a new model was adjusted for each of the outcome variables, taking into account sex, age, and socioeconomic class, as well as the parameters already described. These results were expressed as adjusted odds ratios (OR_adj_) and respective 95%CI. The predictors were considered as fixed factors in the models.

We compared the data on sex, age, socioeconomic class, AUDIT score at baseline, and allocation group among the participants who answered the six-month follow-up *versus* those who did not. This was done to verify if the data of the respondents remained homogeneous regarding the initial sample and to show whether the non-response to the follow-up was different according to the randomization group.

Participants were analyzed in the group for which they were randomized at baseline, in the so-called intention-to-treat (ITT) analysis. We also performed a reanalysis of the data using the Last Observation Carried Forward (LOCF) method; that is, for the participants who did not respond the six-month follow-up, we considered the response at baseline.

Throughout the statistical analysis, we adopted a significance level of 5%.

### Ethics

The study was approved by the Research Ethics Committee of the Universidade Federal de São Paulo (Process 21477), conducted in 2013 and 2014 and registered in the REBEC (Brazilian Registries of Clinical Trials, Ministry of Health), as RBR-35bkzc.

## RESULTS

Of the 1,832 nightclub patrons invited to respond to the online questionnaire sent by e-mail, 610 (33.3%) did not respond (Figure 1). Of this total, 1,057 (86.5%) accepted to participate in the RCT, of which 465 (44.0%) had an AUDIT score greater than or equal to eight – “problem drinkers” – and were then randomized into two groups: intervention (n = 224) and control (n = 241).

Thus, 465 patrons (44.0%, 95%CI 41.0–47.0) were classified as “problem drinkers”. Of these, 344 (74.0%, 95%CI 69.7–77.9) and 256 (55.2%, 95%CI 50.5–59.7) individuals responded “yes” for BD_month_ and BD_week_, respectively. Approximately 50% of these problem drinkers reported a lack of control over drinking behavior (n = 231, 95%CI 45.1–54.4).

According to Table 1, the intervention and control groups were homogeneous regarding the variables of age (p = 0.237), sex (p = 0.099), ABEP socioeconomic status (p = 0.852), and AUDIT score (p = 0.332).

**Table 1:** Distribution of the 465 participants according to group, sociodemographic data, and AUDIT score at baseline.

Variable		Total (n = 465)		Group				t	p
				Intervention (n = 224)		Control (n = 241)			
		n	%	n	%	n	%		
Age (years)								-1.265	0.237
	Average (SD)	24.7 (6.0)		24.3 (5.7)		25.0 (6.2)			
	Minimum-Maximum	18–55		18–50		18–55			
Sex								2.729	0.099
	Male	300	64.5	136	60.7	164	68.0		
	Female	165	35.5	88	39.3	77	32.0		
ABEP								3.744	0.852
	A	139	29.9	68	30.4	71	29.5		
	B	261	56.1	123	54.9	138	57.2		
	C, D, or E	65	14.0	33	14.7	32	13.3		
AUDIT score								-1.050	0.332
	Average (SD)	12.6 (4.1)		12.4 (4.0)		12.8 (4.2)			
	Minimum-Maximum	8–33		8–27		8–33			
AUDIT								0.940	0.639
	Risk use	333	71.6	164	73.2	169	70.1		
	Harmful use	100	21.5	47	21.0	53	22.0		
	Dependence	32	6.9	13	5.8	19	7.9		

SD: standard deviation; t: chi-square value or t-test; ABEP: Brazilian Association of Population Studies; AUDIT: Alcohol Use Disorders Identification Test

At the six-month follow-up, we obtained the response of 79 (50.6%) participants from the intervention group and 77 (49.4%) participants from the control group. Table 2 shows the attrition results of the sociodemographic data, AUDIT score at baseline, and allocation group among these 156 respondents *versus* nonresponders. There was no statistically significant difference between participants who answered the questionnaire after six months *versus* nonresponders.

**Table 2:** Distribution of 465 party goers according to the response to the six months of follow-up.

Variable		Total (n = 465)		Participants (n = 156)		Losses (n = 309)		t	p
		N	%	n	%	n	%		
Group								0.573	0.449
	Intervention	224	48.2	79	50.6	145	46.9		
	Control	241	51.8	77	49.4	164	53.1		
Age (years)								0.171	0.865
	Average (SD)	24.7 (6.0)		24.7 (5.4)		24.6 (6.2)			
	Minimum-Maximum	18–55		18–50		18–55			
Sex								0.005	0.942
	Male	300	64.5	101	64.7	199	64.4		
	Female	165	35.5	55	35.3	110	35.6		
ABEP								0.954	0.621
	A	139	29.9	51	32.7	88	28.5		
	B	261	56.1	85	54.5	176	57.0		
	C, D, or E	65	14.0	20	12.8	45	14.5		
Initial AUDIT_score_								-0.678	0.542
	Average (SD)	12.6 (4.1)		12.4 (4.1)		12.7 (4.1)			
	Minimum-Maximum	8–33		8–27		8–33			
Initial _AUDIT_								0.111	0.940
	Risk use	333	71.6	113	72.5	220	71.2		
	Harmful use	100	21.5	33	21.1	67	21.7		
	Dependence	32	6.9	10	6.4	22	7.1		

SD: standard deviation; t: chi-square value or t-test; ABEP: Brazilian Association of Population Studies; Initial AUDIT: Initial Alcohol Use Disorders Identification Test

Table 3 presents the summary measures for the outcomes evaluated at baseline and after six months of follow-up, according to group. We can observe that there was no significant difference between the two groups in either of the two moments of evaluation. We also observed no significant effect of the intervention after six months of follow-up in any of the evaluated outcomes. However, the OR of the intervention effect at six months for the outcomes evaluated are less than 1, showing that the intervention group presented a decrease in BD_month_, BD_week_, and lack of control when compared to the control group.

**Table 3:** Comparisons between groups in both times and intervention effect in six months (group and time interaction) for ITT and LOCF analysis.

		Groups													
Variable		Intervention (n = 224)			Control (n = 241)			Comparison between groups^a^				Intervention effect in six months^b^			
		n	%	95%CI	n	%	95%CI	OR_c_ a	95%CI	OR_adj_ b	95%CI	OR_c_ a(95%CI)	p	OR_adj_ b(95%CI)	p
Intention-to-treat – ITT														
BD_month_															
	Baseline	161	71.9	66.0–77.8	183	75.9	70.5–81.3	0.81	0.53–1.23	0.79	0.52–1.20	0.97 (0.48–1.94)	0.930	0.98 (0.49–1.98)	0.959
	Six months	51	63.9	53.3–73.8	54	69	59.0–79.0	0.78	0.41–1.49	0.77	0.40–1.48				
BD_week_															
	Baseline	119	53.4	46.5–59.9	137	56.8	50.6–63.1	0.87	0.60–1.25	0.91	0.63–1.31	0.92 (0.52–1.66)	0.795	0.92 (0.51–1.67)	0.794
	Six months	32	41.8	31.8–51.8	34	47.2	37.0–57.4	0.80	0.45–1.44	0.84	0.46–1.51				
Lack of control															
	Baseline	111	49.8	43.2–56.3	120	49.8	43.5–56.1	0.99	0.69–1.44	1.01	0.69–1.45	0.56 (0.30–1.03)	0.062	0.55 (0.30–1.02)	0.058
	Six months	35	43.9	33.6–54.2	49	58.4	48.1–68.7	0.56	0.30–1.01	0.55	0.30–1.01				
Last Observation Carried Forward – LOCF															
BD_month_															
	Baseline	161	71.9	66.0–77.8	183	75.9	70.5–81.3	0.81	0.53–1.23	0.79	0.52–1.20	0.98 (0.75–1.29)	0.901	0.99 (0.74–1.31)	0.928
	Six months	161	68.3	62.2–74.4	183	73	67.4–78.6	0.80	0.53–1.19	0.78	0.52–1.16			
BD_week_															
	Baseline	119	53.4	46.8–59.9	137	56.8	50.6–63.1	0.87	0.60–1.25	0.91	0.62–1.31	0.94 (0.76–1.17)	0.591	0.94 (0.75–1.17)	0.580
	Six months	111	49.8	43.2–56.3	132	54.8	48.5–61.0	0.82	0.57–1.18	0.85	0.59–1.23				
Lack of control															
	Baseline	111	49.8	43.2–56.3	120	49.8	43.5–56.1	0.99	0.69–1.44	1.00	0.69–1.45	0.90 (0.72–1.12)	0.352	0.90 (0.71–1.13)	0.352
	Six months	106	47.5	41.0–54.1	121	50.2	43.9–56.5	0.90	0.62–1.29	0.90	0.62–1,30				

ITT: intention-to-treat analysis; LOCF: last observation carried forward; BD_month_: binge drinking in the month; BD_week_: binge drinking in the week

a Reference: control group.

b Generalized linear model with Stata xtlogit procedure adjusted by group effect, time, group*time, sex, age, and ABEP. Reference: control group.

In addition, we can note a marginally significant result (p = 0.058) for the intervention effect in reducing the prevalence of lack of control over drinking behavior (OR_adj_ = 0.55, 95%CI 0.30–1.02). However, when data are imputed via LOCF, there is no trend of significance for the intervention effect on lack of control over drinking.

**Figure 2:**
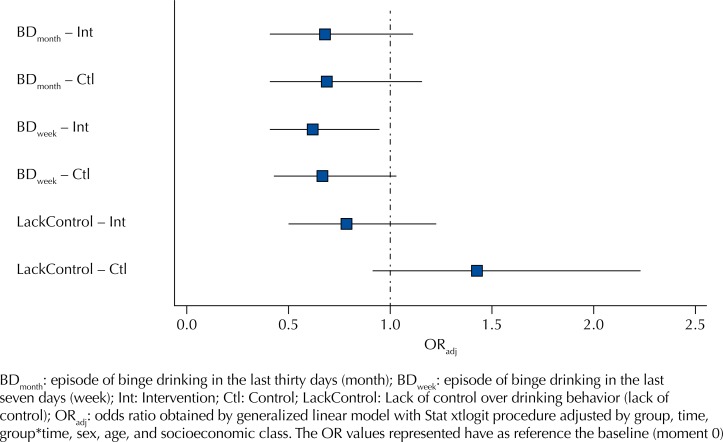
Forest plot for the comparison of intragroup proportion changes, comparing baseline (0) and six months of follow-up, for each outcome evaluated. (n = 465)

Figure 2 shows the comparison of the time outcomes for each group evaluated. There was a significant effect of the intervention group for BD_week_, that is, we observed a reduction of 38% (p = 0.026) in the sixth month of follow-up in the practice of binge drinking in the week, after adjusting for sex, age, and socioeconomic class, when compared to baseline; for the control group, no significant effect was observed (p = 0.062) for this same outcome.

## DISCUSSION

The results presented here are part of the first epidemiologic research in Brazil on risk behavior in parties in the city of São Paulo, Brazil. The study investigated the effect of an online intervention after six months to reduce the practice of BD among nightclub patrons. The main finding was the reduction in 38% of the report of BD_week_ after six months of exposure to the intervention. The group of problem drinkers accounted for 44% of the respondents in the parties of the city and, therefore, require immediate intervention or treatment^30^. The results also showed that most subjects who chose to participate in the RCT were male (64.5%) and belonged to the socioeconomic classes A or B (86%). Average age was 24.7 years.

The sociodemographic data of the participants of the intervention corroborate the data of Siliquini et al.^7^, who have found a proportion of 67.9% of men, aged between 20 and 24 years, and with high education level, suggesting good socioeconomic class, in nightclubs in six European countries. Another Brazilian study, conducted in Belo Horizonte, State of Minas Gerais, with 913 persons who had left leisure environments such as bars and parties, has found a population of 80% of males, aged between 18 and 30 years, and with a family income greater than eight minimum wages^35^.

Web-based interventions based on custom normative feedback are most commonly applied to college students^13^. Kypri et al.^19^, in a study with 2,435 New Zealand university students who were problem drinkers according to the AUDIT classification, have found a reduction of 9% in the frequency of drinking, 6% in the drinking amount, and 14% in the volume consumed after six months of exposure to a web-based intervention of personalized normative feedback. On the other hand, in a systematic review, Foxcroft et al.^36^ have evaluated 66 studies seeking to determine the reduction of the harmful consequences of alcohol abuse in students from normative feedback. There were no significant benefits associated with interventions with social norms in this population, but the authors emphasize the difficulty of comparing these studies given the heterogeneity of design^36^.

In the city of Curitiba, in State of Paraná, a study with a university population comparing web-based interventions with non-computerized interventions (based on motivational interviews) has found more positive results in web-based interventions in the reduction of alcohol use, recommending this alternative to personal interviews, since they are easily accessed by students and cover a greater number of participants^24^.

This type of tool seems to have positive effects when analyzing the general population. In a systematic review, Tait and Christensen^9^ have analyzed fourteen studies (n = 7,082) regarding online interventions for young persons with problem drinking and they have found a reduction of 12% in the amount of alcohol consumed, 35% in the frequency of BD, and 57% in the negative consequences of alcohol use in the different studies. In Brazil, Andrade et al.^23^ have also analyzed digital tools in the general population in a study with 929 participants. The authors emphasize that, despite the relatively low adherence to the study, alcohol consumption can be reduced after six weeks in 62.5% of the users with harmful alcohol consumption, emphasizing the advantage of the tools being available to remote populations and at any time of the day.

The main advantage of the use of digital tools is their accessibility, especially among young university students^16^, a population that largely makes up the scenario of parties in São Paulo^32^, besides their cost-effectiveness and practicality^15^. For Simon-Arndt et al., who have applied the same type of intervention in US seamen, another great advantage of the method was the privacy provided by the Internet when compared to face-to-face actions^17^.

In this study, there was a significant reduction in the practice of BD in the week estimated at 38% among participants in the intervention group after six months, not observed in the control group. However, when evaluating the effect of the program (group-time interaction), the results for BD are not significant and the reduction in the lack of control is marginally significant, even though all odds ratios show a protective trend for the intervention. The more severe option, from a statistical point of view, of using the LOCF approach, considering that the losses maintained their baseline consumption pattern, suggests that there is no intervention effect in any of the evaluated outcomes. Thus, we cannot categorically state that said intervention is effective in this scenario, since we had a complete loss of significance when we included the losses in the analyses from the worst possible scenario (no change).

Among the limitations of this study, we can mention the rate of loss of subjects at the different stages, the difficulty of access to the Internet of some party goers, limiting the amplitude of the RCT, and the impossibility of comparing our results with other studies, given the unprecedented nature of the use of web-based interventions among nightclub patrons. Another limitation is related to the adaptation of the criterion of BD for women, since in this study, we considered as BD five or more doses for both sexes, while several studies choose to reduce the amount to four doses for women. Even with these limitations, the innovative nature of this study is reinforced in Brazil, a country marked by great damages from the abuse of alcohol by the population.

More studies are needed to evaluate this and other methodologies that reduce the prevalence and consequences of alcohol abuse, given the impossibility to reach a conclusion about the effectiveness of web-based intervention tools in reducing BD among nightclub patrons.
